# The Role of Chemokines in Shaping the Balance Between CD4^+^ T Cell Subsets and Its Therapeutic Implications in Autoimmune and Cancer Diseases

**DOI:** 10.3389/fimmu.2015.00609

**Published:** 2015-11-30

**Authors:** Nathan Karin, Gizi Wildbaum

**Affiliations:** ^1^Department of Immunology and Rappaport Family Institute for Research in the Medical Sciences Rappaport Faculty of Medicine, Technion – Israel Institute of Technology, Haifa, Israel

**Keywords:** chemokines, T cell subsets, EAE, CXCR3, CXCL11, CXCL10, cancer, immunotherapy

## Abstract

Chemokines are the key activators of adhesion molecule and also drivers of leukocyte migration to inflammatory sites and are therefore mostly considered as proinflammatory mediators. Many studies, including ours, imply that targeting the function of several key chemokines, but not many others, could effectively suppress inflammatory responses and inflammatory autoimmunity. Along with this, a single chemokine named CXCL10 could be used to induce antitumor immunity, and thereby suppress myeloma. Our working hypothesis is that some chemokines differ from others as aside from being chemoattractants for leukocytes and effective activators of adhesion receptors that possess additional biological properties making them “driver chemokines.” We came up with this notion when studying the interlay between CXCR4 and CXCL12 and between CXCR3 and its three ligands: CXCL9, CXCL10, and CXCL11. The current mini-review focuses on these ligands and their biological properties. First, we elaborate the role of cytokines in directing the polarization of effector and regulatory T cell subset and the plasticity of this process. Then, we extend this notion to chemokines while focusing on CXCL 12 and the CXCR3 ligands. Finally, we elaborate the potential clinical implications of these studies for therapy of autoimmunity, graft-versus-host disease, and cancer.

## Introduction

Chemokines are small (~8–14 kDa), secreted proteins, structurally similar to cytokines that regulate cell trafficking through interactions with a subset of seven-transmembrane G protein-coupled receptors (GPCRs) (1–3). Aside from attracting leukocytes to sites of inflammation, chemokines are tightly involved in the activation of adhesion molecules to allow leukocyte extravasation (4–8). This makes them key drivers of inflammation. Studies coming from our laboratory also imply that aside from chemoattraction, some of these chemokines are involved in directing the polarization of CD4^+^ T cell subsets. This includes the balance between effector T cells subsets (9–11) as well as directing the polarization of effector TH1/Th17 cells into IL-10 producing Tr1-like cells (9–12). The current review focuses on these findings and their biological significance.

## Cytokines That Regulate the Balance Between CD4^+^ T Cells Subsets as Drivers and Regulators of Inflammation

Cytokines are involved in the induction of inflammatory responses by two different, yet complementary, mechanisms: the first includes a direct effect aimed at destructing invading microbes. Two cytokines that posses a major function in this function are tumor necrosis factor alpha (TNF-α) and IL-1β. Consequently, during inflammatory autoimmunity, they are thought to be key mediators of the harmful anti-self distractive response and are, therefore, major targets for therapy of these diseases (13–16). The other mechanism includes directing the functional development (polarization) of CD4^+^ T cells subsets, and thereby the dynamics of the inflammatory process. The notion that the cytokine milieu at the site of inflammation drives T-cell polarization came from early studies showing that while IL-12 skews the TH1/TH2 balance into IFN-γ^high^ IL-4^low^ TNFα producing TH1 cells, IL-4 shifts this balance toward IFN-γ^low^ IL-4^high^ TH2 cells, capable of restraining the inflammatory activities of TH1 cells (17–20). Along with this notion, Leonard et al. showed that blocking IL-12 inhibits experimental autoimmune encephalomyelitis (EAE) by shifting the TH1/Th2 balance toward TH2 (21). Another cytokine that has been associated with shifting the TH1/TH2 balance toward TH1 is IL-18 (IGIF) (22). Following this publication, we observed that target neutralization of this cytokine suppresses autoimmunity by interfering in the TH1/TH2 balance toward TH2 (23), and also that targeted expression of its natural inhibitor, IL-18 binding protein (24) at also suppress the disease by the same mechanism (25). A major concern in applying therapies aiming at shifting the TH1/TH2 balanced toward TH2 is that the last are also a subtype of effector T cells that promote IL-4-dependent immunity (26). Thus, shifting anti-self immunity from TH1 to TH2 might result in an unexpected form of self-destructive immunity (27).

In 2005, IL-17-expressing T cells (TH17 cells) were proposed to be a third, independent TH-cell lineage with a role in inflammatory and autoimmune diseases (28). The key cytokines that drive the polarization of these cells vary between rodents and human. In mice, IL-6 together with transforming growth factor-beta (TGF-β) are likely to induce TH17 at early stages of its polarization (together with IL-21) followed by stabilization by IL-23 (29), whereas in human the combination of IL-1 and IL-6, but not TGF-β are key drivers of TH17 polarization (30). More recently, it has been proposed that TH17 cells may also hold anti-inflammatory properties due to potential expression of CD39 and CD73 ectonucleotidases, leading to adenosine release and the subsequent suppression of CD4^+^ and CD8^+^ T cell effector functions (31).

The activity of effector T cells is tightly regulated by regulatory T cells that fall into two major subtypes, those expressing the master forkhead box protein 3 (FOXP3) that has a major role in directing their biological properties (32). They suppress the activities of effector T cells and of inflammatory macrophages by various mechanisms, thus maintaining self-tolerance (33–36). Aside from nTregs, FOXP3-positive T cells could be polarized from FOXP3-negative T cells (*in vitro*) in the presence of transforming growth factor β (TGF-β) (37).

In 1997, Maria Grazia Roncarolo and her coworkers discovered the reciprocal FOXP3-negative IL-10^high^-producing Tr1 cells (38) that also play a major part in maintenance of self-tolerance (39). These cells could be polarized *in vitro* by either IL-10 + IL-2 (38) or by the combination of IL-10 + Rapamycin (40) and in human by IL-10 + IFNα (41).

## Cytokines and the Plasticity of CD4^+^ T Cell Subsets

First evidence for potential plasticity in CD4^+^ T cell subsets have been demonstrated by Anderson et al. in 2007 showing that during chronic cutaneous leishmaniasis TH1 may gain the Tr1-like phenotype and largely produce IL-10 (42). It is not known if these IL10^high^ cells are indeed Tr1 cells, or just IL10^high^-producing CD4^+^ T cells, at that time, biomarkers that could distinguish Tr1 cell from other IL10^high^ CD4^+^ T cells were not yet identified (41, 43). Later IL-27, together with TGFβ, could repolarize TH1 cells into Tr1 (43, 44). As for FOXP3^+^ Tregs, Chen et al. have shown that coculturing with TGFβ may transform FOXP3^−^CD4^+^ T cells into FOXP3^+^ Tregs, also known as induced Tregs (iTregs) (37). The stability of iTregs *in vivo* is still questionable.

More recent studies focused on the plasticity between TH17 cells and FOXP3^+^ Tregs. It appears that expression of Foxp3 by iTreg cells or IL-17 by Th17 cells may not be stable and that there is a great degree of flexibility in their differentiation options as they emerge from an overlapping developmental program (45). Much of the attention has been devoted to exploring the transition from TH17 to iTregs, though a very recent study showed that the inflammatory environment in autoimmune arthritis induces conversion of a subset of Foxp3^+^ T cells into interleukin-17-producing cells that contribute to disease pathogenesis (46).

These findings should be taken into consideration in designing future therapies aiming at redirecting the polarization of T cell subsets.

## The Role of Chemokines in Driving the Functional Development (Polarization) of CD4^+^ T Cell Subset, are There “Driver” Chemokines?

Chemokines are small (~8–14 kDa), structurally related chemotactic cytokines that regulate cell trafficking through interactions with specific seven-transmembrane, GPCRs (1–3). One of the important features of GPCRs is their ability to transmit diverse signaling cascades upon binding different ligands (47–51). This large family of related molecules is classified on the basis of structural properties, regarding the number and position of conserved cysteine residues, to give two major (CXC and CC) and two minor (C and CX_3_C) chemokine subfamilies (1–3) (Table 1).

**Table 1:** **Key chemokine receptors and their ligands**.

Receptor	Ligands
**CXCR and their ligands**	
CXCR1	CXCL6, CXCL8
CXCR2	CXCL1, CXCL2, CXCL3, CXCL5, CXCL6, CXCL7, CXCL8, (MIF?)
CXCR3	CXCL9, CXCL10, CXCL11
CXCR4	CXCL12, (MIF?)
CXCR5	CXCL13
CXCR6	CXCL16
CXCR7	CXCL11, CXCL12
**CCR**	
CCR1	CCL3, CCL5, CCL7, CCL14, CCL15, CCL16, CCL23
CCR2	CCL2, CCL7, CCL8, CCL13
CCR3	CCL2, CCL5, CCL7, CCL8, CCL11, CCL13, CCL15, CCL24, CCL26, CCL28
CCR4	CCL17, CCL22
CCR5	CCL3, CCL4, CCL5, CCL7, CCL11, CCL13
CCR6	CCL20
CCR7	CCL19, CCL21
CCR8	CCL1
CCR9	CCL25
CCR10	CCL27, CCL28

Most of the attention has been drawn to the key role of these chemotactic mediators in promoting lymphocyte migration processes critical for the onset of inflammatory processes with a special interest in inflammatory autoimmune diseases. Reviewing the results of the very many studies in which single chemokines or there receptors were targeted reviles a major paradox; even though most of the 50 known chemokines can direct the migration of the same leukocytes, targeted neutralization of only one chemokine, such as CCL2, CCL3, CCL5, or CXCL10, is sufficient to suppress the entire inflammatory process (10, 52–59). Therefore, the question that begs an answer is why other chemokines that also attract the same type of leukocyte to the autoimmune site do not compensate for the absence of this single chemokine. In addition, it is also not clear why neutralization of as few as eight to 10 of the 50 different chemokines can effectively suppress the attacks in autoimmune inflammatory diseases (10, 52–59). Hence, what are the attributes of this limited number of chemokines that make them so important in the regulation of inflammatory processes?

A partial explanation for this paradigm could be that these chemokines might have other biological actions that are associated with these autoimmune inflammatory diseases. This includes directing the mobilization of various cells types from the bone marrow to the blood, and later their colonization at the inflammatory site, induction of selective migration to specific organs, directing the development cell subtypes (such as CD4^+^ T cell polarization) or potentiation of innate immune cells. The current review focuses on the role of chemokines on the balance of T cell subsets. CXCL10 is a key driver of TH1 and possibly TH17 polarization and has, therefore, been a major target for neutralization in different autoimmune diseases (9, 10). More recently, we identified two different CXC chemokines that possess anti-inflammatory properties (11, 12).

CXCL12 is an important chemokine that participates in the regulation of tissue homeostasis, immune surveillance, cancer development, and the regulation of inflammatory responses. It is believed that under non-inflammatory conditions, the continuing expression of CXCL12 in tissues that are partially segregated from the immune system, such as the CNS, is important for directing the entry of leukocytes to these sites, as part of immune surveillances (60, 61). We have previously shown that aside from this activity, which in its nature could be proinflammatory, CXCL12 also drives the polarization of CXCR4^+^ macrophages into the IL-10^high^ M2c-like macrophages (12) that hold anti-inflammatory properties (62) and also of effector CD4^+^ T cells (CXCR4^+^) into IL-10^high^ Tr1 cells (12). This may explain why its administration during late stages of EAE leads to rapid remission (12). Based on the above, we thought of generating an Ig-based stabilized protein (CXCL12-Ig) for therapy of various inflammatory autoimmune diseases. Nevertheless, the major involvement of this chemokine in various biological functions, such as homing of stem cells to the bone marrow, homeostasis of neutrophils, angiogenesis, and others precludes it use as a stabilized chemokine for therapy of autoimmune diseases (63).

## CXCL11 as a Novel Driver Anti-Inflammatory Chemokine

Is CXCL12 an exception, or are there other chemokine with anti-inflammatory properties?

One of the important features of GPCRs is their ability to transmit diverse signaling cascades upon binding different ligands (47–51). The Nobel prizewinner Robert J. Lefkowitz and his team have previously raised the concept that different ligands binding the same G-coupled receptor may induce diverse signaling cascades resulting in distinct biological activities (50, 64, 65). Even though the mechanistic basis of this feature is not fully understood, its biological and clinical implications are highly significant (50).

**Figure 1:**
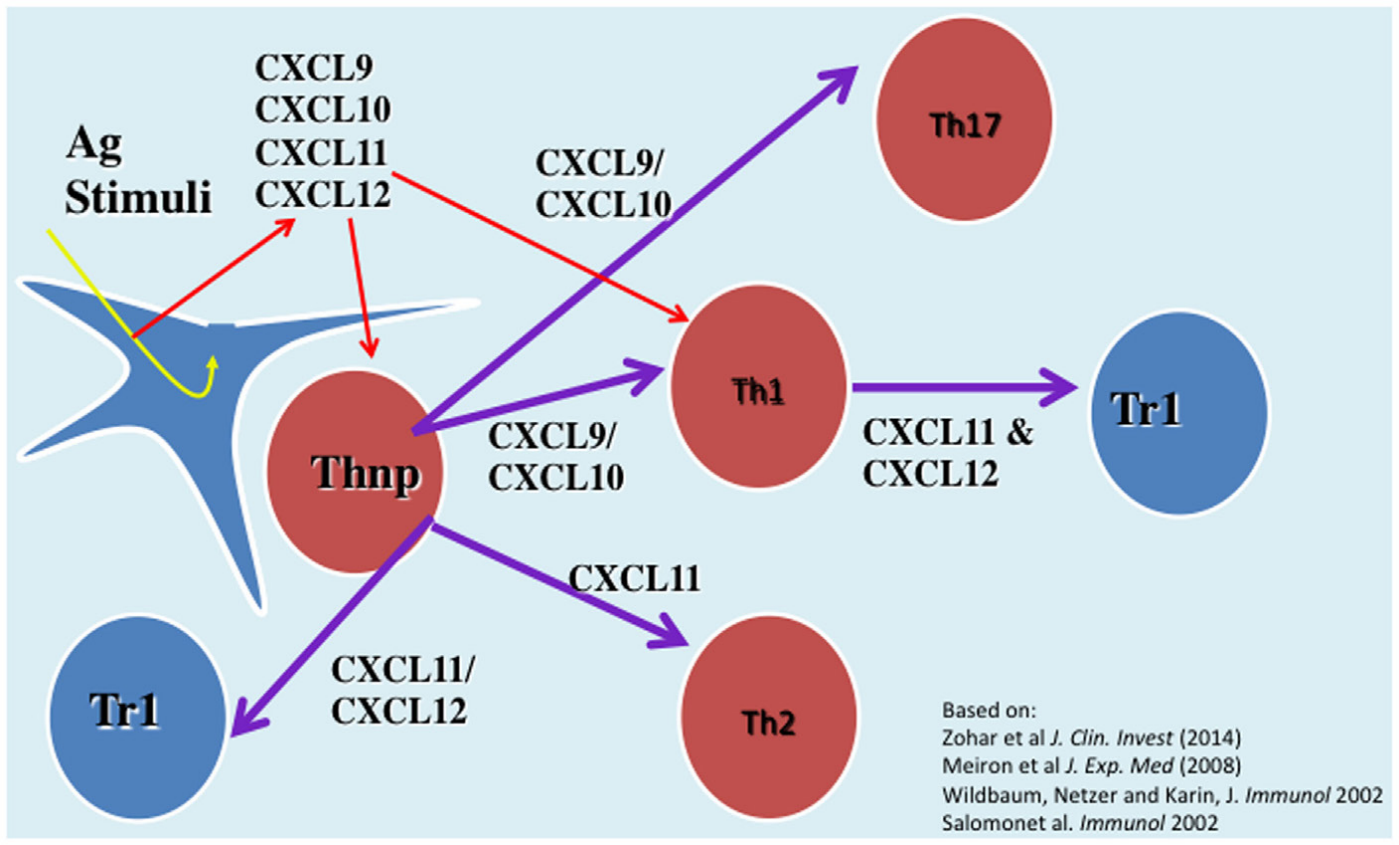
**The role of CXC chemokines in driving the polarization and biological function of CD4^+^ T cell subsets**.

We have investigated the interplay between CXCR3 and its three ligands: CXCL9, CXCL10, and CXCL11 on directing the polarization of CD4^+^ T cells. We observed that while CXCL9 and CXCL10 skew T cell polarization into Th1/Th17 effector cells, CXCL11 drives CD4^+^ T cell polarization into IL-10-producing Tr-1 (11). We also uncovered the signaling basis of this biased response, and learned that it is GαI independent (11). While CXCL10/CXCR3 interactions drive effector Th1 polarization via STAT1, STAT4, and STAT5 phosphorylation, CXCL11/CXCR3 binding induces an immunotolerizing state that is characterized by IL-10^high^ (Tr1) and IL-4^high^ (Th2) cells and mediated via p70 kinase/mTOR in STAT-3- and STAT-6-dependent pathways (11). CXCL11 binds CXCR3 a higher affinity than CXCL10, suggesting that CXCL11 has potential to mediate and restrain inflammatory autoimmunity (Figure 1). This may explain, in part, why CXCR3-deficient mice develop an extremely severe form of EAE and T1DM (66, 67).

## Novel Approach for Chemokine-Based Therapy of Inflammatory Autoimmunity GVHD and Cancer Diseases

Thus, far many affords has been spent in studying exploring the therapeutic potential of targeting the interaction between chemokine and their receptors for treating various autoimmune and cancer diseases. This includes antibody-based therapy to single chemokines or their receptors (68, 69), targeted DNA vaccines that that amplify the natural autoantibody titer to chemokines (9, 10, 55, 70), soluble chemokine receptor-based therapy (71, 72), and small molecule-based antagonists to chemokine receptors (73, 74). Some of these studies have been employed in human clinical trials, thus far with very limited successes. It is believed that the major limitation of applying anti-chemokine- or chemokine receptor-based therapies is the redundancy between chemokines and the enhanced *in vivo* production, once being neutralized (71). The discovery of chemokines with anti-inflammatory properties opens the door for an alterative approach of using stabilized chemokines for therapy of autoimmunity and graft-versus-host disease (GVHD).

Could stabilized chemokines be also used for therapy of cancer diseases? Studies that were initiated in experimental models and recently extended to patients suffering from melanoma showed that blockage of FOXP3^+^ T cells function by blocking the interaction between immunosuppressive receptor programmed cell death-1 (PD-1) largely expressed on FOXp3^+^ T cells and its target coreceptor on antigen presenting cells (PDL-1) using anti-PD1 mAb (nivolumab) (75) or anti-PDL-1 mAb (76) suppressed the function of tumor infiltrating Tregs, and thereby enhanced antitumor immunity to suppress tumor development and progression (75, 76). The other successful approach of enhancing antitumor immunity against melanoma included the administration of a mAb (ipilimumab) which blocks cytotoxic T-lymphocyte-associated antigen 4 (CTLA-4) to potentiate an antitumor T-cell response (77). Very recently, combined therapy\ of anti-PD1 (nivolumab) and anti-CTLA-4 (ipilimumab) showed improved efficacy in treating melanoma (78). The observations that CXCL10 enhances effector T cell activities (11) motivated us to explore CXCL10-Ig-based therapy in cancer diseases. Very recently, we showed that indeed administration of CXCL10-Ig in a clinical set-up of myeloma that CXCL10-Ig could be used for immunotherapy of this disease, and that aside from enhancing antitumor immunity, it directly suppresses tumor growth (79). Along with this study, very recently, Barreira da Silva et al. showed that inhibition of DPP4 enzymatic activity enhanced tumor rejection by preserving biologically active CXCL10 and increasing trafficking into the tumor by lymphocytes expressing the counter-receptor CXCR3 (80). We are now exploring combined therapies of CXCL10-Ig with anti-PD1 or anti-CTLA-4 in a melanoma set-up.

Another chemokine that might serve as a target for cancer therapy is CCL1. Its CCR8 receptor is highly expressed on FOXP3^+^ Tregs and has been associated in their targeted attraction (81, 82). Along with this, Hoelzinger et al. showed that targeting CCL1 might enhance antitumor immunity (83). We are now examining whether its stabilized form (CCL1-Ig) could be used for therapy of inflammatory autoimmunity.

## Conclusion

The current review focuses on exploring the involvement of chemokines in directing the polarization and biological function of CD4^+^ T cells. Thus, far most of the attention has been devoted to exploring the role of cytokines in this property. From a clinically oriented perspective, the findings that chemokines may also polarize Tregs (so far our data shows relevance only for FOXP3-negative Tregs) opens the window of opportunities for using stabilized chemokines for therapy of inflammatory autoimmunity and GVHD, and also for cancer diseases. The basic rational is that the stabilized form of chemokines that induce Tr1-like cells, among them CXCL12 and CXCL11, could be used for therapy of autoimmunity and GVHD, whereas stabilized CXCL10 would be used for cancer therapy.

We find some major differences between CXCL12 and CXCL11 as potential tolerizing chemokines. CXCL12 also renders anti-inflammatory properties in macrophages (12), whereas CXCL11 also polarizes IL-4^high^ Th2 cells (11). We assume that CXCL11 could be a better candidate for being a potential drug since CXCL12 is involved in many biological activities aside from being an immunoregulator, such as neutrophil homeostasis or stem cell homing (63).

## Ethics Statement

All experimental work described in the manuscript was approved by the ethical committee of the Technion, according the NIH guideline.

## Conflict of Interest Statement

The authors declare that the research was conducted in the absence of any commercial or financial relationships that could be construed as a potential conflict of interest.
